# An Autopsy Report of Beta-Propeller Protein-Associated Neurodegeneration with 68-Year Survival, Focusing on Isoform-Specific Distribution of Hyperphosphorylated Tau

**DOI:** 10.3390/reports9030209

**Published:** 2026-07-01

**Authors:** Tomonori Kai, Keiko Tominaga, Atsumi Matsunaga, Hiroshi Shimizu, Kazuhiro Iwama, Keisuke Ishizawa

**Affiliations:** 1 Department of Pathology, Tokyo Metropolitan Tama Medical Center, Fuchu 183-8524, Japan; 2 Department of Internal Medicine, Tokyo Metropolitan Fuchu Medical Center for the Disabled, Fuchu 183-8553, Japan; 3 Healthcare Center, Irumagawa Hospital, Saitama 350-1305, Japan; 4 Department of Pathology, Brain Research Institute, Niigata University, Niigata 951-8122, Japan; hshimizu@bri.niigata-u.ac.jp; 5 Department of Human Genetics, Yokohama City University Graduate School of Medicine, Yokohama 236-0004, Japan; kiwama@yokohama-cu.ac.jp; 6 Department of Pediatrics, Yokohama City University Hospital, Yokohama 236-0004, Japan; 7 Department of Laboratory Medicine (Neuropathology), Tokyo Metropolitan Neurological Hospital, Fuchu 183-0042, Japan; ishizawa@saitama-med.ac.jp; 8 Department of Pathology, Saitama Medical University, Saitama 350-0495, Japan

**Keywords:** beta-propeller protein-associated neurodegeneration, BPAN, static encephalopathy of childhood with neurodegeneration in adulthood, SENDA, neurodegeneration with brain iron accumulation, NBIA, *WDR45*, autophagy, tauopathy

## Abstract

**Background and Clinical Significance**: Beta-propeller protein–associated neurodegeneration (BPAN), also known as static encephalopathy of childhood with neurodegeneration in adulthood (SENDA), is a subtype of neurodegeneration with brain iron accumulation caused by pathogenic variants in *WDR45*. Although its clinical course and neuroimaging features are increasingly recognized, detailed neuropathological characterization, especially at its terminal stage, remains limited. **Case presentation**: We report a 68-year-old woman with a heterozygous *WDR45* splice-site variant (NM_007075.4:c.830+1G>A), representing the longest-surviving case of SENDA/BPAN described to date. After static developmental delay in childhood, she rapidly developed progressive parkinsonism, dystonia, and cognitive decline in early adulthood, ultimately becoming bedridden with profound motor and autonomic dysfunction. Serial MRI demonstrated progressive cerebral and cerebellar atrophy with iron-related signal changes in the globus pallidus and substantia nigra. She died of sepsis at the age of 68 and was subjected to an autopsy including the brain. **Neuropathological findings**: Autopsy revealed severe, diffuse neuronal loss and gliosis throughout the central nervous system, with marked iron deposition and complete neuronal loss in the globus pallidus and substantia nigra. Immunohistochemistry demonstrated widespread tau pathology. Notably, neuronal tau inclusions contained both four-repeat (4R) and three-repeat (3R) isoforms, whereas glial tau was predominantly 4R-positive, indicating a mixed neuronal 4R/3R and glial 4R-dominant tauopathy. Perivascular and subpial 4R-tau–dominant deposits consistent with aging-related tau astrogliopathy were also present. LC3-positive and ferritin-positive cells suggested impaired autophagic flux, supporting the proposed autophagy-related pathogenesis of SENDA/BPAN. **Conclusions**: This case provides comprehensive clinicopathological insight into end-stage SENDA/BPAN, highlighting distinctive tau isoform patterns in neurons versus glia and pathological evidence of autophagy dysfunction. These findings expand the neuropathological spectrum of SENDA/BPAN and may inform future mechanistic and therapeutic research.

## 1. Introduction and Clinical Significance

Beta-propeller protein-associated neurodegeneration (BPAN) [1], also known as static encephalopathy of childhood with neurodegeneration in adulthood (SENDA) [2,3], is an entity of neurodegeneration with brain iron accumulation (NBIA). The prevalence of this condition remains unclear, though it has been reported to affect approximately two to three per million individuals [4]. Pathogenic variants in *WDR45* are now known to be responsible for the disease [1,2,5]. *WDR45* is located on the X chromosome, and according to previous literature, approximately 81–92% of cases are caused by de novo mutations [6,7]. Because *WDR45* resides on the X chromosome, one report indicates that approximately 85% of affected individuals are female [7], while male patients are reported to exhibit somatic mosaicism [5,7,8]. Recent studies revealed the pathogenesis associated with autophagy and iron metabolism [9]. Furthermore, it is also known as a form of mixed four-repeat (4R) and three-repeat (3R) tauopathy [1,10]. While its clinical course and brain imaging have been extensively characterized, detailed neuropathological characterization, especially at its terminal stage, remains limited.

Herein we report an autopsied case of SENDA/BPAN with the longest survival so far reported, where drastic neuropathological changes in the central nervous system (CNS), including tau composition and distribution, are described, in addition to some insight into the pathogenesis related to autophagy and iron metabolism.

## 2. Case Presentation

The patient is a 68-year-old woman who died of sepsis from urinary tract infection, with a definite diagnosis of SENDA/BPAN by the presence of the *WDR45* gene variant disclosed when she was 65 years old (y/o) after a period of 35 years of long-term admission for severe motor, mental, and intellectual disabilities. Until 65 y/o, she had been supposed to have an unknown progressive neurological disease of the CNS. She had no family history of movement disorders, dementia, or psychiatric illness. She was born weighing 3460 g in a state of apparent death at the 39th gestational week. Her development course was protracted: acquiring visual tracking at 3 months old (m/o); holding her head up at 5 m/o; starting to walk at 24 m/o; and uttering only one word at 3 y/o. Her intellectual and physical development reached a plateau at 10 y/o, and her cognitive and movement dysfunction did not particularly worsen for the next 15 years. However, at 26 y/o, the muscle tonus of her extremities began to become more rigid, and gait disturbance reminiscent of parkinsonism began to become more recognized. Then within the next only 4 years, she eventually became bedridden and ceased to utter a word, with her face being “mask-like.” Around 35 years of age, her posture was consistent with decorticate rigidity. Her voluntary movements completely disappeared at 58 y/o.

With the progress of her disease, she had many complications such as repeated aspiration pneumonias, repeated urinary tract infections, paralytic ileus, and CO_2_ narcosis. After tube feeding and artificial respiration management, however, eventually at 60 y/o, she was managed with intravenous hyperalimentation.

**Figure 1:**
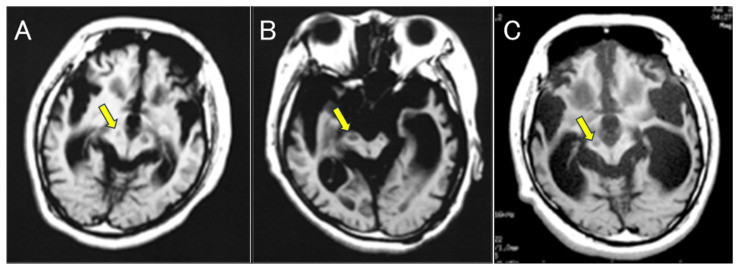
T1-weighed imaging at 36 y/o, 43 y/o, and 50 y/o, respectively, demonstrates progressive cerebral atrophy and hyperintensity of the substantia nigra with a central area of hypointensity (arrows) (**A**–**C**).

**Figure 2:**
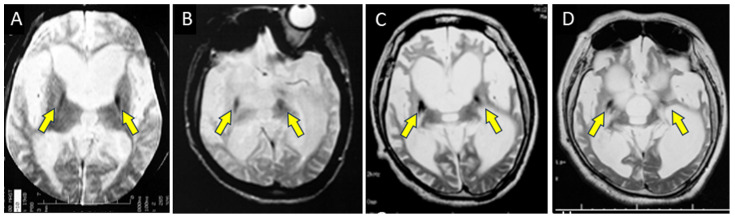
T2-weighed imaging demonstrates hypointensity of the globus pallidus (arrows) at 36 y/o, 43 y/o, 50 y/o, and 58 y/o, respectively (**A**–**D**).

A total of four brain magnetic resonance imaging (MRI) scans were performed during her long clinical course. The first brain MRI was performed at 36 y/o, where T1-weighted imaging (T1WI) showed hyperintensity with a central area of hypointensity in the substantia nigra (SN) (Figure 1A), and T2-weighted imaging (T2WI) showed hypointensity in the globus pallidus (GP) (Figure 2A). Another round of brain MRI was performed thereafter, three times in all (43 y/o, 50 y/o, and 58 y/o). These T1WI (Figure 1B,C) and T2WI (Figure 2B–D) showed similar signal intensity in the SN and GP to the first one, but cerebral and cerebellar atrophy became more prominent. (Figure 1A–C). Seen in a retrospective manner, these neuroradiologic features were consistent with those of SENDA/BPAN [2,11]; however, her diagnosis as SENDA/BPAN remained unestablished even with these brain MRI findings because it was much later in 2012 that SENDA/BPAN was proposed as a new type of NBIA.

**Figure 3:**
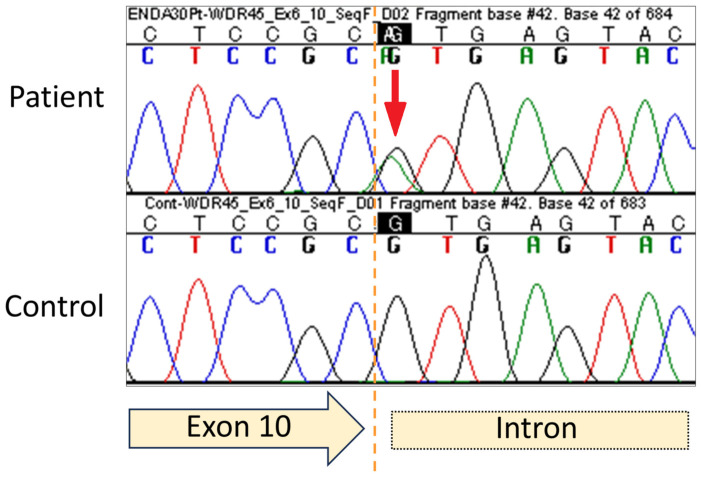
Direct sequence analysis of *WDR45* shows heterogeneous substitution c830+1G>A (red arrow) in this patient. This variant is splicing site variant, leading to aberrant splicing. This variant in *WDR45* was already reported for SENDA/BPAN. Blue lines indicate cytosine, red lines indicate thymine, black lines indicate guanine, and green lines indicate adenine.

When she was 65 y/o, considering her symptoms that had acutely worsened in her twenties coupled with the results of past brain MRI, a diagnosis of SENDA/BPAN was highly suspected. A molecular genetic analysis of *WDR45* using genomic DNA in her blood was performed, where a direct sequence analysis identified a heterogeneous substitution (c830+1G>A) at the splicing site in *WDR45* (splicing substitution NM_007075.3), which is a pathogenic variant due to aberrant splicing (Figure 3). This variant is one of those already reported for *WDR45* in SENDA/BPAN. The molecular genetic analysis of *WDR45* for her parents was not performed because they had already passed away.

She died of sepsis from urinary tract infection at the age of 68, and an autopsy including the brain was performed.

Informed consent for autopsy and possible publication was obtained from the family of the patient. The study was conducted in accordance with the Declaration of Helsinki and approved by the ethics committee of Tokyo Metropolitan Fuchu Medical Center for the Disabled and Yokohama City University Faculty of Medicine.

## 3. Autopsy Findings

### 3.1. Gross Examination of the Central Nervous System (CNS)

**Figure 4:**
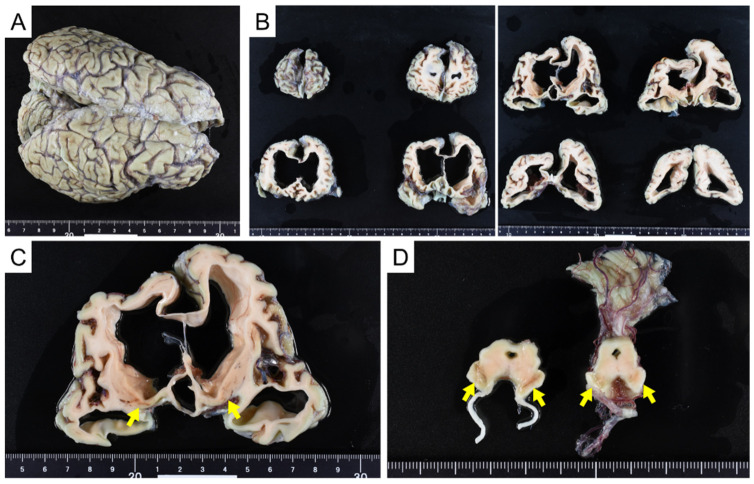
Gross findings of the brain. (**A**) Diffuse atrophy of the cerebrum, especially in the frontal lobes. (**B**) Coronal sections showing severe thinning of gray and white matter with marked enlargement of the lateral and third ventricles. (**C**,**D**) Brownish pigmentation is observed bilaterally in the GP (arrows, (**C**)) and SN (arrows, (**D**)). (GP, globus pallidus; SN, substantia nigra).

The brain weighed 516 g and was diffusely softened. Atrophy was diffuse, but it was particularly prominent in the frontal lobes (Figure 4A).

On coronal sections (Figure 4B), the brain showed severe thinning of gray and white matter with enlargement of the lateral and third ventricles.

Brownish pigmentation was bilaterally observed in the GP and SN, accompanying severe atrophy of these nuclei (Figure 4C,D).

The spinal cord was severely and diffusely atrophic.

Tumors, inflammation, congestion, or foci of hemorrhage or infarction were not observed.

### 3.2. Microscopic Examination of the CNS

On hematoxylin and eosin (HE) sections, the GP and SN showed the most remarkable pathologic alterations.

**Figure 5:**
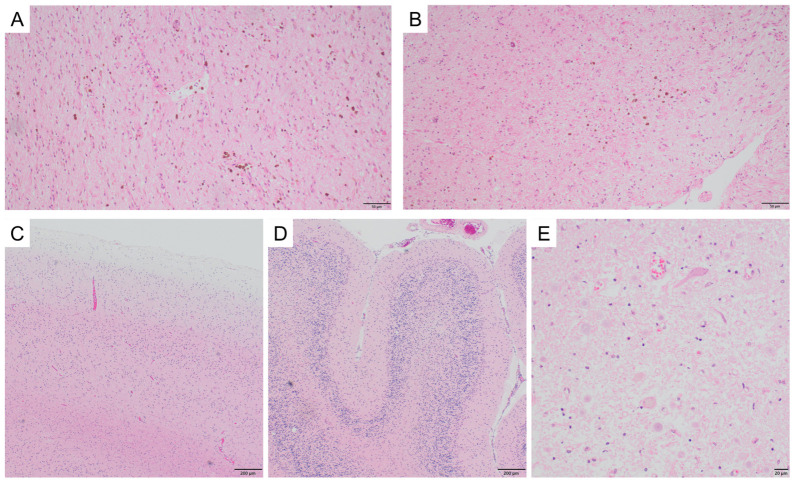
Histological findings. (**A**,**B**) Iron deposits with severe neuronal loss and gliosis in the GP (**A**) and SN (**B**). (Scale bars = 50 μm, original magnification ×200). (**C**,**D**) Severe neuronal loss and gliosis in the cerebral (**C**) and cerebellar (**D**) cortices. (Scale bars = 200 μm, original magnification ×20). (**E**) Neuronal loss and gliosis in the anterior horn of C7. (Scale bar = 20 μm, original magnification ×200).

The GP (Figure 5A) and SN (Figure 5B) showed severe neuronal loss and gliosis with prominent hemosiderosis. The iron granules were identified in the brain parenchyma as well as around the vessels (i.e., Virchow–Robin space). They were largely deposited intracellularly, such as within astrocytes and microglia/macrophages. A number of round, granular, and eosinophilic structures with or without calcium deposition, which were reminiscent of foamy spheroid bodies (FSB) [12,13,14] or eosinophilic granular bodies (EGB) [15], were also noted in these nuclei. Some of these structures partially or entirely consisted of calcification.

There were no iron deposits in the neuroanatomic structures other than the GP and SN.

The cerebral cortex (Figure 5C) showed marked neuronal loss with or without laminar necrosis, leading to disappearance of six-layered structures, where marked and diffuse gliosis was also observed.

The hippocampus also showed severe neuronal loss both in the cornu ammonis and dentate fascia. Senile plaques were not identified in the hippocampus proper, subiculum, or entorhinal cortex.

The brainstem, including the cerebellar peduncles, was also severely atrophic. The red nucleus showed neuronal loss, and the locus coeruleus also showed neuronal loss with melanin incontinence. The hypoglossal nuclei showed a reduced number of neurons.

The cerebellar cortex (Figure 5D) showed severe loss of Purkinje cells accompanied by Bergmann gliosis.

The spinal cord (Figure 5E) was diffusely atrophic with marked gliosis, particularly in the anterior and lateral funiculi, where some axonal spheroids were present. Inclusions or demyelinating foci were not identified. The neurons in the anterior and posterior horns were reduced in number and often atrophic.

On HE sections throughout, intracytoplasmic or intranuclear inclusions were not identified in neurons or glial cells.

### 3.3. Immunohistochemistry of the CNS

An immunohistochemical study was conducted for further analyses. The primary antibodies used targeted the following antigens: glial fibrillary acidic protein (GFAP) (mouse monoclonal, GA5,1:500, Novocastra (Leica, Wetzlar, Germany)); hyperphosphorylated tau (p-tau) (AT8, mouse monoclonal, IGH135,1:2000, Thermofisher, (Waltham, MA, USA)); amyloid β (Aβ) (mouse monoclonal, 12B2, 1:500, Immuno-Biological Laboratories (IBL, Fujioka, Japan)); p62c (mouse polyclonal, 1:10,000, Abcam, (Cambridge, UK); neurofilament protein (NFP) (mouse monoclonal, 2F11, 1:500, Dako, (Glostrup, Denmark)); neuron-specific nuclear protein (NeuN, mouse monoclonal, A60, 1:2000, Millipore (Merck, Darmstadt, Germany)); three-repeat (3R-) tau (RD3, mouse monoclonal, 8E6/C11,1:1000, Millipore(Merck, Darmstadt, Germany)); four-repeat (4R-) tau (RD4, mouse monoclonal, 1E1/A6,1:100, Millipore (Merck, Darmstadt, Germany)); CD68 (KP-1, mouse monoclonal, ready-to-use, Dako (Glostrup, Denmark)); LC3B(rabbit polyclonal, PM036, 1:5000, MBL (Tokyo, Japan)); and ferritin light chain (goat polyclonal, ab110017, 1:100, Abcam (Cambridge, UK)). After incubation with the primary antibodies, the sections were incubated with a commercialized kit of secondary antibodies (Ventana ultraView DAB universal Kit (Ventana, Basel, Switzerland) or Histofine Simple Stain MAX-PO Kit (Nichirei, Tokyo, Japan)) and then visualized with diaminobenzidine as the chromogen.

**Figure 6:**
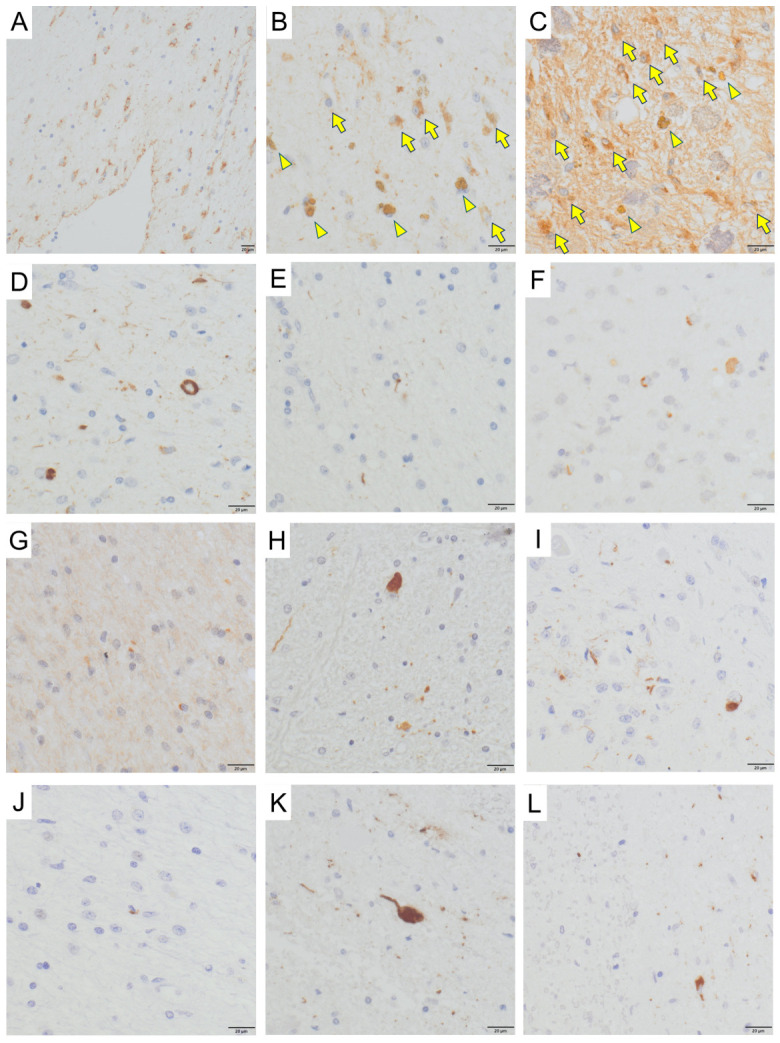
Immunohistochemical results. (**A**) AT8-positive astroglial deposits corresponding to ARTAG in the GP (AT8-immunostaining). (**B**) AT8-positive intracytoplasmic inclusions in glial cells (arrows) in non-perivascular or non-subpial regions. Iron granules are also noted (arrowheads; AT8-immunostaining). (**C**) LC3-positive cells (arrows) and iron granules (arrowheads) in the GP (LC3-immunostaining). (**D**) AT8-positive neuronal deposits and threads in the middle frontal gyrus (AT8-immunostaining). (**E**) AT8-positive glial deposits and threads in the middle frontal gyrus (AT8-immunostaining). (**F**) RD4-positive neuronal and glial deposits in the middle temporal gyrus (RD4-immunostaining). (**G**) RD3-positive neuronal deposits in the middle temporal gyrus (RD3-immunostaining). (**H**) AT8-positive neuronal and glial deposits and threads in the medullary reticular formation (AT8-immunostaining). (**I**) AT8-positive neuronal and glial deposits and threads in the dentate nucleus (AT8-immunostaining). (**J**) AT8-positive glial deposits and threads in the cerebellar white matter (AT8-immunostaining). (**K**) AT8-positive neuronal deposit and threads in the anterior horn of C7 (AT8-immunostaining). (**L**) AT8-positive neuronal deposit and glial deposits in the posterior horn and the lateral funiculus of Th9, respectively (AT8-immunostaining). (**A**) Scale bar = 20 μm; taken at original magnification ×200. (**B**–**L**) Scale bar = 20 μm; taken at original magnification ×400.

The GP and SN showed no NeuN-positive cells, which was in sharp contrast to flourishing GFAP-positive astrocytes/fibrillary gliosis. Occasionally, p-tau (AT8)-positive astrocytes were noted in perivascular or subpial regions, consistent with aging-related tau astrogliopathy (ARTAG) (Figure 6A) [16,17,18,19], which were mostly positive for four-repeat tau (RD4), but not for three-repeat tau (RD3). Besides, especially in the SN, some intracellular glial AT8-positive deposits not necessarily corresponding to ARTAG were found in non-perivascular or non-subpial regions (Figure 6B), which represented an RD4-positive immunophenotype.

There were some glial cells that were positive for LC3 (Figure 6C). The aforementioned FSB- or EGB-like structures were positive for ubiquitin and p62 but negative for NFP and GFAP. Most iron-containing cells were positive for CD68, indicating that they were macrophages.

The cerebral cortices showed diffuse staining for GFAP, disclosing diffuse gliosis. NeuN revealed a reduced number of neurons. Both neurons (Figure 6D) and glial cells (Figure 6E) were positive for AT8; the former mostly corresponded to neurofibrillary tangles (NFTs), and the latter, to astrocytic and oligodendrocytic inclusions. NFTs were positive for both RD4 and RD3 (Figure 6F,G), while glial tau was positive for RD4 but negative for RD3. Perivascular and subpial fuzzy staining for AT8, which was consistent with ARTAG, was also observed. No Aβ deposits were observed in the cerebral cortices.

The hippocampus proper, subiculum, and entorhinal cortex showed neuronal AT8-positive deposits, corresponding to Braak NFT stage II [20], suggesting senile tau pathology. No deposition of Aβ was identified.

The brainstem showed marked GFAP-positive gliosis. AT8-positive cells, including neurons and glia, were frequently observed as well (Figure 6H). Similarly to the cerebral cortices, neuronal tau was positive for RD4 and RD3, while glial tau was positive for RD4 but not for RD3. No Aβ deposits were found.

In the cerebellum, no AT8-positive deposits were identified in the cortex, but some neuronal and glial deposits were found in the deep nuclei and white matter, indicating that these are of neuronal and glial origin (Figure 6I,J).

The spinal cord showed neurons or glial cells containing AT8-positive intracytoplasmic inclusions in the anterior and posterior horns and/or the dorsal and lateral funiculi. (Figure 6K,L)

### 3.4. General Autopsy Findings

Clear cell renal cell carcinoma of the right kidney and multiple adenocarcinomas of the sigmoid colon were incidentally noted. There were no metastases in the body, including the CNS.

The direct cause for the death of the patient was considered to be multiple organ failure from urosepsis.

### 3.5. Semiquantitative Analysis

We performed a semiquantitative analysis of p-tau (AT8)-positive immunoproducts in a wide range of neuroanatomic structures of the CNS. The deposits were semiquantitatively graded as follows: rare (±), occasional (+), and frequent (++). The RD4- and RD3-positive immunoproducts were similarly graded as well (Table 1).

## 4. Discussion

NBIA is a group of clinically and genetically defined neurodegenerative diseases, characterized by extrapyramidal movement disorder, intellectual deterioration, and characteristic deposition of iron in the basal ganglia [8,11,21].

SENDA/BPAN is classified as a subtype of NBIA, and mutated *WDR45* was reported as the causative gene [2,5]. One of the clinical features of SENDA/BPAN is non-specific developmental delays without regression in childhood. Then, in early adulthood, the patients present with progressive parkinsonism, dystonia, spasticity, and intellectual deterioration resulting in severe disability [2].

In our case, the patient was diagnosed as having SENDA/BPAN by a heterozygous substitution in *WDR45* (c.830+1G>A) at 65 years old. The variant substitute of guanine with adenine at the +1 position of the canonical splice donor site located at the boundary of exon 10 and intron 10 is known to disrupt splicing machinery. According to the variant classification framework established by the American College of Medical Genetics and Genomics (ACMG) [22], variants affecting the ±1 or ±2 positions of canonical splice sites constitute strong evidence of a loss-of-function mechanism (PVS1; pathogenic very strong). Given that this variant has been independently reported in multiple unrelated cases [5,23,24,25] (PS4; pathogenic strong), it is classified as pathogenic.

Until 26 years of age, her clinical feature had been the static developmental delay, and in early adulthood, the motor and intellectual disorders rapidly progressed. Then she became bedridden with mental deterioration. Even after becoming bedridden, her deterioration continued and resulted in loss of voluntary movements and autonomic dysfunctions, such as paralytic ileus and neurogenic bladder.

To the best of our knowledge, this is the case of SENDA/BPAN with the longest survival in the medical literature, wherein the clinical and pathological features of SENDA/BPAN at the terminal stage, when the physical and mental deterioration has fully progressed, are supposed to be well displayed and sufficiently recognizable. Marked neuronal loss and severe gliosis over the entire CNS are considered to reflect the incessantly progressing clinical course of the disease, although a number of repetitions of hypoxic events should also have contributed to the pathologic alterations at the terminal stage.

SENDA/BPAN is also known as a tauopathy [1], accompanying NFTs, pre-tangles, and threads widely seen over the brain, and both of 4R- and 3R-tau is demonstrated by Western blotting [10]. However, there have been no papers distinguishing between neuronal and glial deposits in previous literature. In this case, we assessed p-tau isoforms by immunohistochemistry and found that the tauopathy due to SENDA/BPAN is a mixed 4R- and 3R-neuronal and a predominantly 4R-glial tauopathy. The difference of isoform composition between neuronal tau and glial tau in SENDA/BPAN is the novel finding.

Pathologically, Alzheimer’s disease (AD) is an important differential diagnosis due to the similar pattern of tau deposition [26,27,28,29]. However, this case presented no senile plaques or Aβ accumulation in the CNS, suggesting that the tau deposits in this case are not due to AD. Primary age-related tauopathy (PART) is another unignorable differential diagnosis. PART typically shows tau accumulation as a form of NFT, predominantly in the medial temporal region and hippocampus. In contrast to tau, there is little Aβ deposit in this disease [29,30,31]. In this case, tau deposits were found diffusely in the cerebrum, brain stem, and cerebellum, and even in the spinal cord. Just as described above, typical PART predominantly involves the medial temporal region and merely minimally affects, if at all, the brainstem or cerebellum. The distribution of tau deposits did not show any preference to the medial temporal region in this case, which is not consistent with PART.

Other differential diagnoses include such primary tauopathies as progressive supranuclear palsy (PSP), corticobasal degeneration (CBD), and globular glial tauopathy (GGT) [17,27,28,29], but the brain in this case did not show any pathologic hallmarks of these disorders, including tufted astrocytes, astrocytic plaques, and globular astrocytic/oligodendrocytic inclusions.

In this case, another pattern of distribution of tau was noted, i.e., the perivascular and subpial 4R-dominant tau deposits. As has already been shown in the Results, this pattern of tau distribution is most consistent with ARTAG [16,17,18,19]. Some previous reports indicate that ARTAG can coexist with other tauopathies like AD, PSP, CBD, and argyrophilic grain disease or non-tauopathy neurodegenerative diseases including Parkinson’s disease [17,18,32]. The present case likely demonstrates that ARTAG can coexist with the tauopathy of SENDA/BPAN, just as in those neurodegenerative diseases.

SENDA/BPAN is a disorder of autophagy processing, interfering with iron recycling and its accumulation in the brain [2,11]. *WDR45* encodes WIPI4, a WD domain-containing autophagy-related protein. WIPI4 plays a role in autophagosome formation by mediating ATG2A/B, which supplies lipids required for the phagophore membrane’s extension, and it is known that lack of WIPI4 function results in size reduction of autophagosome [33]. Thus given, its genetic variants may cause autophagy flux.

Autophagy also contributes to cellular iron homeostasis through ferritinophagy, which works as an autophagic pathway responsible for ferritin degradation [23]. Consequently, loss of functional *WDR45* protein is expected to impair autophagic flux and dysregulate iron metabolism. Although excess iron itself was considered to produce reactive oxygen species (ROS) and lead to neuronal death [20], a recent research [9] noted that pathogenesis of SENDA/BPAN is a shortage of iron (II), which is a physiologically available form of iron, but not an excess of iron (III) that has the potential to produce ROS.

LC3 is a marker of autophagosome [34]. Autophagy processes normally decompose autophagosomes so that LC3-positive autophagosomes would not be either accumulated in the brain or seen on brain tissues. In this case, LC3-positive cells and ferritin-positive cells were seen, potentially reflecting the failure of reduction of iron (III) to iron (II) in autophagosomes. This is consistent with the suggested malfunction of the autophagy operating system in SENDA/BPAN. In this sense, the present study could be a pathological corroboration of the pathogenesis of SENDA/BPAN suggested so far.

Especially in the GP and SN, complete neuronal loss and frequent iron accumulation were demonstrated in the present case. The iron granules were deposited in the parenchyma as well as around the vessels, phagocytosed by macrophages or astrocytes. Additionally, p-tau deposits were identified in these nuclei. Some of them were considered to correspond to ARTAG, as has already been discussed above, but some other p-tau deposits, which were not necessarily consistent with ARTAG, were identified, suggesting that these are p-tau deposits that are highly probably due to SENDA/BPAN itself.

Although the pathogenesis of tauopathy due to SENDA/BPAN remains to be further clarified, it can be assumed that autophagy failure can lead to abnormal tau accumulation. Autophagy is responsible for the clearance of protein aggregates such as Aβ, tau, and synuclein [35]. For tauopathy, several pathways are known to be involved in tau degradation by autophagy flux, and its impairment would contribute to tau propagation [35,36]. Autophagy has been discussed as a possible therapeutic target for SENDA/BPAN [36].

Taken together, the clinical and pathological findings in the present patient likely corroborate theoretical pathogenesis or in vitro findings so far suggested for SENDA/BPAN.

## 5. Conclusions

The present case of SENDA/BPAN, with the longest survival reported in the literature, provided novel findings associated with tau distribution and its isoform composition or malfunction of the autophagy operating system, in addition to several findings that are consistent with the theoretical pathogenesis of the disease thus far reported. Further elucidation of the pathogenesis of SENDA/BPAN and its potential therapeutic approach, however, should await further collection of cases of SENDA/BPAN, especially those at its terminal stage when the natural history of the disease is fully represented, as in the present case.

## Figures and Tables

**Table 1:** Semiquantitative analysis of p-tau distribution in the CNS.

Location		AT8		RD4		RD3	
		Neuronal	Glial	Neuronal	Glial	Neuronal	Glial
**CEREBRUM**	Middle frontal gyrus	++	+	++	+	++	−
	Inferior frontal gyrus	++	+	N/A	N/A	N/A	N/A
	Temporal lobe	+	+	++	++	++	−
	Inferior parietal lobule	++	+	N/A	N/A	N/A	N/A
	Occipital lobe (lingual gyrus)	++	+	N/A	N/A	N/A	N/A
	Insular cortex	++	+	++	+	+	−
	Caudate head	+	+	+	+	+	−
	Putamen	+	+	+	+	+	−
	Globus pallidus	No neurons	+	No neurons	+	No neurons	±
	Thalamus	+	++	−	+	+	−
	Anterior hippocampus	++	++	+	+	+	−
	Posterior hippocampus	++	+	+	+	++	±
**MIDBRAIN**	Substantia nigra	No neurons	++	No neurons	+	No neurons	−
	Red nucleus	+	+	+	+	-	−
	Reticular formation (midbrain)	++	+	+	+	+	−
	Cerebral peduncle	N/A	+	N/A	+	N/A	−
	Superior cerebellar peduncle	N/A	+	N/A	+	N/A	−
**PONS**	Locus coeruleus	++	+	N/A	N/A	N/A	N/A
	Reticular formation (pons)	++	+	N/A	N/A	N/A	N/A
	Pontine nucleus	+	+	N/A	N/A	N/A	N/A
	Middle cerebellar peduncle	N/A	+	N/A	N/A	N/A	N/A
	Longitudinal fiber	N/A	+	N/A	N/A	N/A	N/A
**MEDULLA**	Inferior olivary nucleus	+	+	+	+	+	−
	Hypoglossal nucleus	+	+	−	+	−	−
	Vagal dorsal motor nucleus	++	+	+	+	+	+
	Reticular formation (medulla)	++	+	+	+	+	−
	Inferior cerebellar peduncle	N/A	+	N/A	+	N/A	−
**CEREBELLUM**	Cerebellar cortex	−	+	−	−	−	−
	Dentate nucleus	++	−	N/A	N/A	N/A	N/A
	Cerebellar white matter	N/A	+	N/A	N/A	N/A	N/A
**SPINAL CORD**	Cervical (C7, 8)	+	+	N/A	N/A	N/A	N/A
	Thoracic (Th9, 10)	+	+	N/A	N/A	N/A	N/A

−, none; +, occasional; ++, frequent; N/A, not available/applicable; C, cervical; Th, thoracic.

## Data Availability

The data that support the findings of this study are available from the corresponding author upon reasonable request.

## Abbreviations

The following abbreviations are used in this manuscript:

BPAN	beta-propeller protein–associated neurodegeneration
SENDA	static encephalopathy of childhood with neurodegeneration in adulthood
4R	four-repeat
3R	three-repeat
NBIA	neurodegeneration with brain iron accumulation
CNS	central nervous system
MRI	magnetic resonance imaging
T1WI	T1-weighted imaging
T2WI	T2-weighted imaging
SN	substantia nigra
GP	globus pallidus
HE	hematoxylin and eosin
FSB	foamy spheroid bodies
EGB	eosinophilic granular bodies
GFAP	glial fibrillary acidic protein
Aβ	amyloid β
p-tau	hyperphosphorylated tau
NFP	neurofilament protein
NeuN	neuron-specific nuclear protein
ARTAG	aging-related tau astrogliopathy
NFT	neurofibrillary tangle
PART	primary age-related tauopathy
AD	Alzheimer’s disease
PSP	progressive supranuclear palsy
CBD	corticobasal degeneration
ROS	reactive oxygen species

## References

[1] Hayflick S.J., Kruer M.C., Gregory A., Haack T.B., Kurian M.A., Houlden H.H., Anderson J., Boddaert N., Sanford L., Harik S.I. (2013). Beta-Propeller Protein-Associated Neurodegeneration: A New X-Linked Dominant Disorder with Brain Iron Accumulation. Brain.

[2] Saitsu H., Nishimura T., Muramatsu K., Kodera H., Kumada S., Sugai K., Kasai-Yoshida E., Sawaura N., Nishida H., Hoshino A. (2013). De Novo Mutations in the Autophagy Gene *WDR45* Cause Static Encephalopathy of Childhood with Neurodegeneration in Adulthood. Nat. Genet..

[3] Kimura Y., Sato N., Sugai K., Maruyama S., Ota M., Kamiya K., Ito K., Nakata Y., Sasaki M., Sugimoto H. (2013). MRI, MR Spectroscopy, and Diffusion Tensor Imaging Findings in Patient with Static Encephalopathy of Childhood with Neurodegeneration in Adulthood (SENDA). Brain Dev..

[4] Wilson J.L., Gregory A., Kurian M.A., Bushlin I., Mochel F., Emrick L., Adang L., Group B.G.C.A., Hogarth P., Hayflick S.J. (2021). Consensus Clinical Management Guideline for Beta-Propeller Protein-Associated Neurodegeneration. Dev. Med. Child Neurol..

[5] Haack T.B., Hogarth P., Kruer M.C., Gregory A., Wieland T., Schwarzmayr T., Graf E., Sanford L., Meyer E., Kara E. (2012). Exome Sequencing Reveals De Novo *WDR45* Mutations Causing a Phenotypically Distinct, X-Linked Dominant Form of NBIA. Am. J. Hum. Genet..

[6] Mou C., Zhou L., Xiong J.J., Lei L. (2025). A Unique Case of Neurodevelopmental Disorders and Epilepsy Linked to *WDR45* Variant Inheritance and Maternal Mosaicism. Gene.

[7] Stige K.E., Gjerde I.O., Houge G., Knappskog P.M., Tzoulis C. (2018). Beta-Propeller Protein-Associated Neurodegeneration: A Case Report and Review of the Literature. Clin. Case Rep..

[8] Gregory A., Kurian M.A., Wilson J., Hayflick S., Adam M.P., Bick S., Mirzaa G.M., Pagon R.A., Wallace S.E., Amemiya A. (1993). Neurodegeneration with Brain Iron Accumulation Disorders Overview. GeneReviews^®^.

[9] Tsukida K., Muramatsu S., Osaka H., Yamagata T., Muramatsu K. (2022). *WDR45* Variants Cause Ferrous Iron Loss Due to Impaired Ferritinophagy Associated with Nuclear Receptor Coactivator 4 and WD Repeat Domain Phosphoinositide Interacting Protein 4 Reduction. Brain Commun..

[10] Paudel R., Li A., Wiethoff S., Bandopadhyay R., Bhatia K., De Silva R., Houlden H., Holton J.L. (2015). Neuropathology of Beta-Propeller Protein Associated Neurodegeneration (BPAN): A New Tauopathy. Acta Neuropathol. Commun..

[11] Kruer M.C., Boddaert N., Schneider S.A., Houlden H., Bhatia K.P., Gregory A., Anderson J.C., Rooney W.D., Hogarth P., Hayflick S.J. (2012). Neuroimaging Features of Neurodegeneration with Brain Iron Accumulation. Am. J. Neuroradiol..

[12] Arai N., Honda Y., Amano N., Yagishita S., Misugi K. (1988). Foamy Spheroid Bodies in the Substantia Nigra: Report of an Unusual Case with Recurrent Attacks of Peculiar Twilight State. J. Neurol..

[13] Arai N., Yagishita S., Misugi K., Oda M., Kosaka K., Mizutani T., Morimatsu Y. (1992). Peculiar Axonal Debris with Subsequent Astrocytic Response (Foamy Spheroid Body): A Topographic, Light Microscopic, Immunohistochemical and Electron Microscopic Study. Virchows Arch. A Pathol. Anat. Histopathol..

[14] Arai N., Mizutani T., Morimatsu Y. (1993). Foamy Spheroid Bodies in the Globus Pallidus and the Substantia Nigra Pars Reticulata: An Investigation on Regional Distribution in 56 Cases without Neurodegenerative Diseases. Virchows Arch. A Pathol. Anat. Histopathol..

[15] Murayama S., Bouldin T.W., Suzuki K. (1992). Immunocytochemical and Ultrastructural Studies of Eosinophilic Granular Bodies in Astrocytic Tumors. Acta Neuropathol..

[16] Kovacs G.G., Robinson J.L., Xie S.X., Lee E.B., Grossman M., Wolk D.A., Irwin D.J., Weintraub D., Kim C.F., Schuck T. (2017). Evaluating the Patterns of Aging-Related Tau Astrogliopathy Unravels Novel Insights Into Brain Aging and Neurodegenerative Diseases. J. Neuropathol. Exp. Neurol..

[17] Kovacs G.G., Xie S.X., Lee E.B., Robinson J.L., Caswell C., Irwin D.J., Toledo J.B., Johnson V.E., Smith D.H., Alafuzoff I. (2017). Multisite Assessment of Aging-Related Tau Astrogliopathy (ARTAG). J. Neuropathol. Exp. Neurol..

[18] Kovacs G.G., Xie S.X., Robinson J.L., Lee E.B., Smith D.H., Schuck T., Lee V.M.-Y., Trojanowski J.Q. (2018). Sequential Stages and Distribution Patterns of Aging-Related Tau Astrogliopathy (ARTAG) in the Human Brain. Acta Neuropathol. Commun..

[19] Kovacs G.G. (2020). Astroglia and Tau: New Perspectives. Front. Aging Neurosci..

[20] Braak H., Braak E. (1991). Neuropathological Stageing of Alzheimer-Related Changes. Acta Neuropathol..

[21] Wang Z.-B., Liu J.-Y., Xu X.-J., Mao X.-Y., Zhang W., Zhou H.-H., Liu Z.-Q. (2019). Neurodegeneration with Brain Iron Accumulation: Insights into the Mitochondria Dysregulation. Biomed. Pharmacother..

[22] Richards S., Aziz N., Bale S., Bick D., Das S., Gastier-Foster J., Grody W.W., Hegde M., Lyon E., Spector E. (2015). Standards and Guidelines for the Interpretation of Sequence Variants: A Joint Consensus Recommendation of the American College of Medical Genetics and Genomics and the Association for Molecular Pathology. Genet. Med. Off. J. Am. Coll. Med. Genet..

[23] Cong Y., So V., Tijssen M.A.J., Verbeek D.S., Reggiori F., Mauthe M. (2021). *WDR45*, One Gene Associated with Multiple Neurodevelopmental Disorders. Autophagy.

[24] Hornemann F., Le Duc D., Roth C., Pfäffle R., Huhle D., Merkenschlager A. (2020). Childhood Dystonia-Parkinsonism Following Infantile Spasms-Clinical Clue to Diagnosis in Early Beta-Propeller Protein-Associated Neurodegeneration. Neuropediatrics.

[25] Chard M., Appendino J.P., Bello-Espinosa L.E., Curtis C., Rho J.M., Wei X.-C., Al-Hertani W. (2019). Single-Center Experience with Beta-Propeller Protein-Associated Neurodegeneration (BPAN); Expanding the Phenotypic Spectrum. Mol. Genet. Metab. Rep..

[26] Taniguchi-Watanabe S., Arai T., Kametani F., Nonaka T., Masuda-Suzukake M., Tarutani A., Murayama S., Saito Y., Arima K., Yoshida M. (2016). Biochemical Classification of Tauopathies by Immunoblot, Protein Sequence and Mass Spectrometric Analyses of Sarkosyl-Insoluble and Trypsin-Resistant Tau. Acta Neuropathol..

[27] Chung D.C., Roemer S., Petrucelli L., Dickson D.W. (2021). Cellular and Pathological Heterogeneity of Primary Tauopathies. Mol. Neurodegener..

[28] Fiock K.L., Hook J.N., Hefti M.M. (2023). Determinants of Astrocytic Pathology in Stem Cell Models of Primary Tauopathies. Acta Neuropathol. Commun..

[29] Olfati N., Shoeibi A., Litvan I. (2022). Clinical Spectrum of Tauopathies. Front. Neurol..

[30] Kim D., Kim H.-S., Choi S.-M., Kim B.C., Lee M.-C., Lee K.-H., Lee J.-H. (2019). Primary Age-Related Tauopathy: An Elderly Brain Pathology Frequently Encountered during Autopsy. J. Pathol. Transl. Med..

[31] Hickman R.A., Flowers X.E., Wisniewski T. (2020). Primary Age-Related Tauopathy (PART): Addressing the Spectrum of Neuronal Tauopathic Changes in the Aging Brain. Curr. Neurol. Neurosci. Rep..

[32] Koga S., Zhou X., Murakami A., Fernandez De Castro C., Baker M.C., Rademakers R., Dickson D.W. (2022). Concurrent Tau Pathologies in Frontotemporal Lobar Degeneration with TDP-43 Pathology. Neuropathol. Appl. Neurobiol..

[33] Shimizu T., Tamura N., Nishimura T., Saito C., Yamamoto H., Mizushima N. (2023). Comprehensive Analysis of Autophagic Functions of WIPI Family Proteins and Their Implications for the Pathogenesis of β-Propeller Associated Neurodegeneration. Hum. Mol. Genet..

[34] Shojaei S., Barzegar Behrooz A., Cordani M., Aghaei M., Azarpira N., Klionsky D.J., Ghavami S. (2025). A Non-fluorescent Immunohistochemistry Method for Measuring Autophagy Flux Using MAP1LC3/LC3 and SQSTM1 as Core Markers. FEBS Open Bio.

[35] Hamano T., Enomoto S., Shirafuji N., Ikawa M., Yamamura O., Yen S.-H., Nakamoto Y. (2021). Autophagy and Tau Protein. Int. J. Mol. Sci..

[36] Watanabe Y., Taguchi K., Tanaka M. (2020). Ubiquitin, Autophagy and Neurodegenerative Diseases. Cells.

